# Recombinase Polymerase Amplification Combined with Lateral Flow Strip for Rapid Detection of OXA-48-like Carbapenemase Genes in Enterobacterales

**DOI:** 10.3390/antibiotics11111499

**Published:** 2022-10-28

**Authors:** Phatsarawadee Hemwaranon, Arpasiri Srisrattakarn, Aroonlug Lulitanond, Patcharaporn Tippayawat, Ratree Tavichakorntrakool, Lumyai Wonglakorn, Jureerut Daduang, Aroonwadee Chanawong

**Affiliations:** 1 Centre for Research and Development of Medical Diagnostic Laboratories, Faculty of Associated Medical Sciences, Khon Kaen University, Khon Kaen 40002, Thailand; 2 Clinical Microbiology Unit, Srinagarind Hospital, Khon Kaen University, Khon Kaen 40002, Thailand

**Keywords:** carbapenem resistance, Gram-negative bacilli, isothermal amplification

## Abstract

Carbapenem-resistant Enterobacterales (CRE) possessing various carbapenemases, particularly the OXA-48 group, are now rapidly spreading and becoming a major public health concern worldwide. Phenotypic detection of OXA-48-like carbapenemases is still suboptimal due to their weak carbapenemase activity, whereas highly sensitive and specific polymerase chain reaction (PCR)-based methods take at least 3–4 h. We, therefore, developed a recombinase polymerase amplification (RPA) combined with lateral flow (LF) strip assay for the rapid detection of *bla*_OXA-48-like_ in Enterobacterales. A total of 131 clinical isolates including 61 *bla*_OXA-48-like_-carrying Enterobacterales isolates and 70 Gram-negative bacilli isolates containing other *bla* genes were subjected to the RPA method performed under isothermal conditions at 37 °C within 10 min and visually inspected by LF strip within 5 min. The RPA-LF assay provided 100% sensitivity (95% confidence interval, 92.6–100%) and 100% specificity (93.5–100%) for detecting *bla*_OXA-48-like_ genes from bacterial colonies. Its detection limit was 100 times less than that of the PCR method. This assay is rapid, easy to perform, and provides excellent performance without any special equipment. It may be applied for directly identifying the *bla*_OXA-48-like_ genes in Enterobacterales obtained from blood culture. Rapid identification of carbapenemase types is essential for selecting appropriate antimicrobial options, particularly the β-lactams combined with novel β-lactamase inhibitors.

## 1. Introduction

According to the Centre for Disease Control and Prevention (CDC), members of the order Enterobacterales are important pathogens causing various infectious diseases [1]. Rising antimicrobial resistance in these organisms due to the overuse of antimicrobial agents has also become a major public health concern worldwide. Currently, Enterobacterales clinical isolates are resistant to many available antimicrobials, particularly carbapenems commonly used for the treatment of serious infections. This results in serious illness and increased mortality due to the lack of effective and safe treatment options, and leads to higher healthcare expenses [1,2,3]. One of the main resistance mechanisms to carbapenems among these Enterobacterales is carbapenemase production, particularly KPC (*Klebsiella pneumoniae* carbapenemase), NDM (New Delhi metallo-β-lactamase), IMP (active on imipenem), VIM (Verona integron-encoded metallo-β-lactamase) and OXA-48-like (oxacillinase-48-like) [4]. Genes encoding these enzymes could be transferred among various genera of Enterobacterales via plasmids, leading to their global dissemination [2,4]. Antimicrobial drugs such as colistin or tigecycline have been widely used as drugs of choice for the treatment of infections caused by carbapenem-resistant Enterobacterales (CRE) for both carbapenemase-producing Enterobacterales (CPE) and non-CPE [3,5]. However, both chromosomally- and plasmid-mediated colistin resistance among Enterobacterales has emerged worldwide [6]. Alternative treatments for CPE infections by the combination of β-lactam antibiotics with novel β-lactamase inhibitors, such as ceftazidime–avibactam, meropenem–vaborbactam and imipenem–cilastatin–relebactam, have recently been recommended for specific types of carbapenemases [3,5,7,8]. Therefore, identification of carbapenemase types is useful for the selection of appropriate antimicrobial therapy.

To date, OXA-48-like-producing Enterobacterales clinical isolates have emerged globally, including Europe, North Africa, the Middle East, the Indian subcontinent, Asia and sub-Saharan African countries [9,10]. The OXA-48 carbapenemase group includes OXA-48, OXA-162, OXA-181, OXA-204, OXA-232, OXA-244, OXA-245, OXA-438, OXA-484, OXA-517 and OXA-519. The common variants from most to least are OXA-48, OXA-181, OXA-232, OXA-204, OXA-162 and OXA-244. However, some OXA-48-like enzymes, such as OXA-163, OXA-247 and OXA-405, have no carbapenemase activity [10]. Phenotypic tests for identifying OXA-48-like carbapenemases have drawbacks due to their weak carbapenemase activity and the unavailability of suitable inhibitors [11]. The Carba NP test recommended by Clinical and Laboratory Standards Institute (CLSI) is rapid (2 h), highly sensitive and specific for CPE detection [12], but it has low sensitivity for detecting OXA carbapenemases including OXA-48-like enzymes [13]. CLSI has also recommended the modified carbapenem inactivation method (mCIM) for carbapenemase detection in Enterobacterales and *Pseudomonas aeruginosa*, and the combination of the mCIM with the EDTA-carbapenem inactivation method (eCIM) for distinguishing class B carbapenemases or metallo-β-lactamases (MBLs) from classes A and D carbapenemases in Enterobacterales [12]. Despite their convenience and no requirement of special reagents or equipment, both methods are time-consuming (18–24 h) and false-negative results by the mCIM were also found in OXA-48-like producers [14,15]. Currently, the immunochromatographic test (ICT) is available for rapid detection of OXA-48-like carbapenemases at point-of-care (POC) [16,17,18]. Although it is highly sensitive and specific, it is expensive for low-income countries, and false-negative results in OXA-48-like producers were also reported [17]. Apart from phenotypic tests, molecular techniques including polymerase chain reaction (PCR) method were developed for differentiation of known carbapenemase genes including the *bla*_OXA-48-like_ genes [19]. This method has high sensitivity and specificity, but its turnaround time is at least 3–4 h. Both culture-based and PCR-based methods cannot easily be performed at the POC, thereby delaying treatment and potentially leading to inappropriate antimicrobial therapy. Therefore, simple, rapid and sensitive methods for both phenotypic and genotypic detection of OXA-48-like enzymes are still needed.

Isothermal amplification methods for detecting targeted genes are performed using only a water bath, heating block or incubator for generating a low and constant temperature (e.g., 40–60 °C), thereby requiring less power and eliminating the need for complex systems [20,21,22,23]. A recombinase polymerase amplification (RPA) method developed by Piepenburg et al. [22] combines a low constant temperature between 25 and 42 °C with a short reaction duration of around 10–15 min, making it ideal for POC tests [24]. It comprises three key proteins for DNA amplification: a recombinase, strand-displacement DNA polymerase, and single-stranded DNA-binding proteins (SSBs). The RPA products can be visually inspected by agarose gel electrophoresis (AGE), real-time fluorescent probes, an electrochemical biosensor or a gold nanoparticle-based lateral flow biosensor (LF) [24,25]. Among these detection methods, the LF is a device that can detect DNA products at the POC. The presence of a colored band generated by a specific RPA product can be observed by naked eyes within 5 min. The RPA method in combination with the LF strip (RPA-LF) is simple, and now widely applied to detect and identify various antimicrobial resistance genes [26,27,28,29,30,31]. We, therefore, developed the RPA-LF assay for the rapid detection of *bla*_OXA-48-like_ genes, which could be used as a POC diagnostic tool for patients with bloodstream infections, or for infection control purposes in low-resource laboratories. 

## 2. Results

### 2.1. Detection of bla_OXA__-48__-like_ by RPA-LF Assay

**Figure 1:**
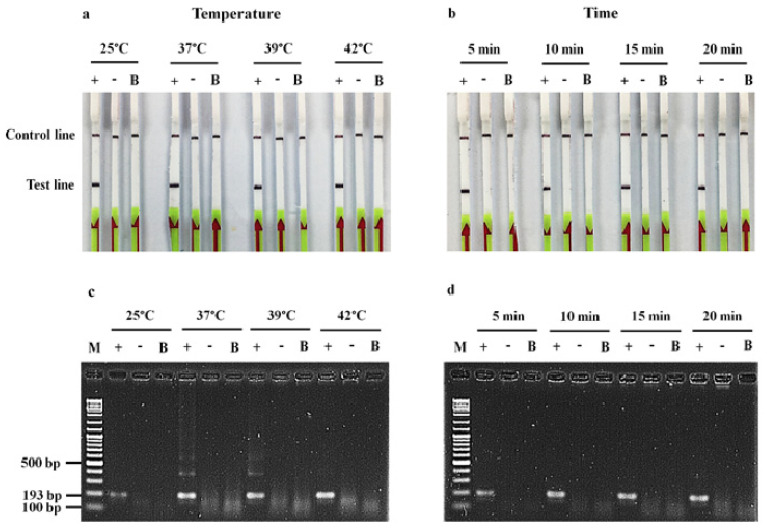
Optimization of the RPA-LF conditions, amplification temperature (**left**) and incubation time (**right**), for identification of the *bla*_OXA__-__48__-__like_. The RPA products were detected by lateral flow strip (**a**,**b**), and agarose gel electrophoresis (**c**,**d**). M, 100 bp DNA ladder; +, *bla*_OXA__-__48__-__like_ positive control strain; -, negative control strain; and B, blank.

The optimization of the RPA-LF assay revealed that the test line of the positive control strain was obvious at any temperature (25, 37, 39 or 42 °C) for 20 min, and any incubation time (5, 10, 15 or 20 min) at 37 °C, whereas the negative control strain showed a visual band of the control line only (Figure 1a,b). Detection of the RPA products by the AGE method provided intense bands for all conditions, except that a faint band was observed after incubation either at 25 °C for 20 min or at 37 °C for 5 min (Figure 1c,d). Therefore, the optimal condition for further RPA-LF experiments was incubation at 37 °C for 10 min.

### 2.2. Detection Limit of RPA-LF, RPA-AGE and PCR-AGE Assays

**Figure 2:**
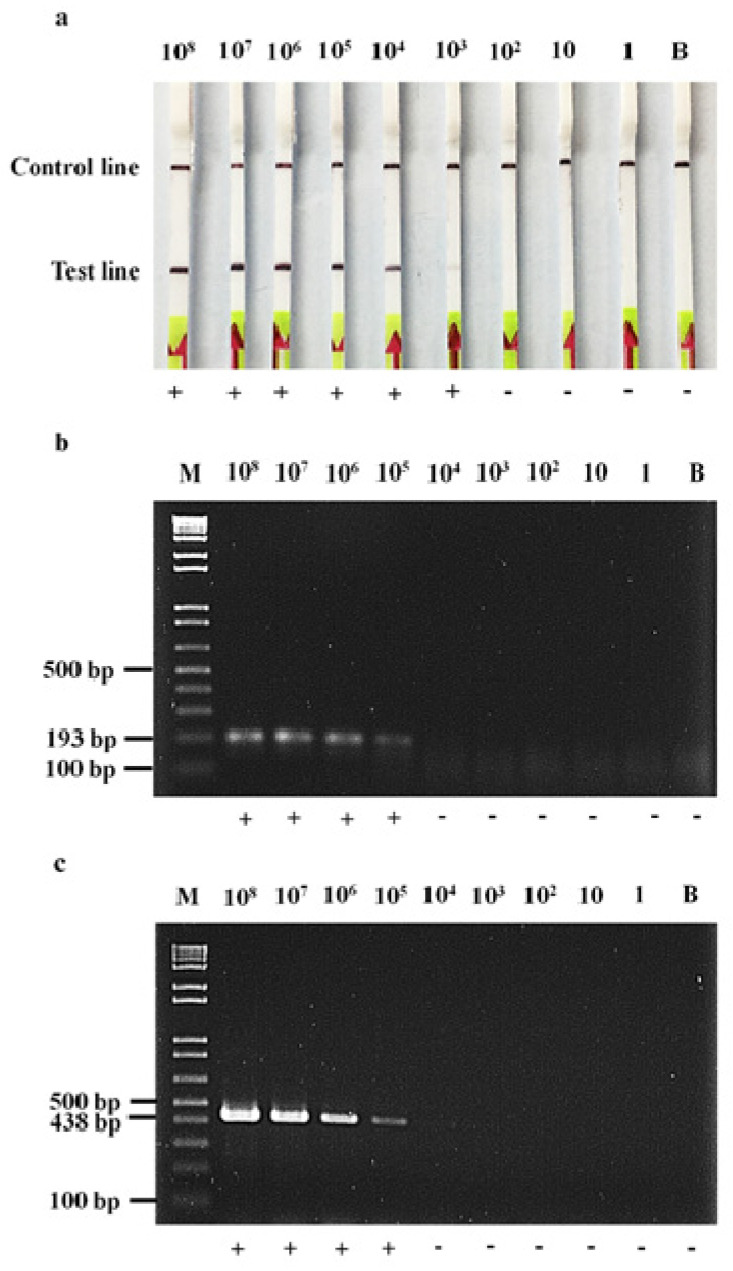
Comparison of the detection limit of the RPA-LF assay (**a**), with assays of the RPA-AGE (**b**), and the PCR-AGE (**c**), for detection of *bla*_OXA-48-like_. M, 1 kb plus DNA ladder; 1-9, 10-fold serial dilutions of *K. pneumoniae* isolates harboring *bla*_OXA-48_ ranging from 10^8^ to 1 CFU/mL, respectively; and B, blank.

The detection limit of the RPA-LF assay for identifying the *bla*_OXA-48-like_ gene was 10^3^ CFU/mL, whereas that of either the RPA-AGE or the PCR-AGE method was 10^5^ CFU/mL (Figure 2).

### 2.3. Assessment of RPA-LF Assay

**Figure 3:**
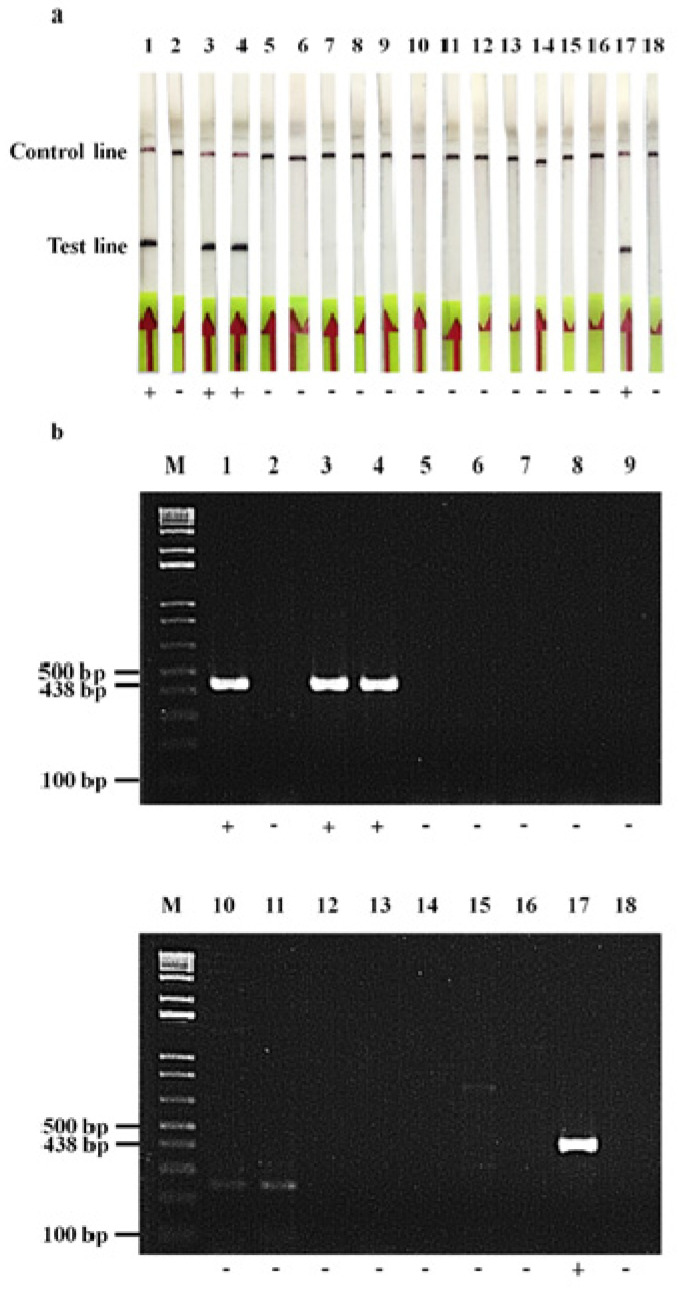
The RPA products of the *bla*_OXA-48-like_ from the reference strains and clinical isolates detected by the RPA-LF (**a**), and PCR-AGE (**b**), assays. M, 1 kb plus DNA ladder; 1, *bla*_OXA-48_ positive control strain; 2, negative control strain; 3, *E. coli* (*bla*_OXA-48-like_); 4, *E. coli* (*bla*_OXA-181_); 5, *K. pneumoniae* ATCC BAA-1705 (*bla*_KPC-2_); 6, *E. cloacae* (*bla*_IMI-1_); 7, *E. coli* (*bla*_NDM_); 8, *K. pneumoniae* (*bla*_IMP-14a_); 9, *P. aeruginosa* (*bla*_VIM-2_); 10, *A. baumannii* (*bla*_OXA-23_); 11, *A. baumannii* (*bla*_OXA-58_); 12, *A. pittii* (*bla*_OXA-72_); 13, *K. pneumoniae* (*bla*_CTX-M-1-like_); 14, *E. coli* (*bla*_CMY-2_); 15, *E. coli* (*bla*_VEB-like_, *bla*_CMY-8b_); 16, *K. pneumoniae* (*bla*_CTX-M-9_, *bla*_SHV-12_, *bla*_VEB-like_); 17, *K. pneumoniae* (*bla*_NDM_, *bla*_OXA-48_); 18, *A. baumannii* (*bla*_NDM_*, bla*_OXA-23-like_, *bla*_OXA-58_).

The RPA-LF assay correctly identified all 61 *bla*_OXA-48-like_-carrying isolates (51 *bla*_OXA-48-like_, 4 *bla*_OXA-48_, 5 *bla*_OXA-181_ and 1 *bla*_OXA-48_ plus *bla*_NDM_), whereas the 53 isolates with other carbapenemase genes (23 *bla*_NDM_, 3 *bla*_IMP-14a_, 2 *bla*_IMP_, 3 *bla*_VIM-2_, 1 *bla*_VIM_, 2 *bla*_IMI-1_, 1 *bla*_IMI_, 1 *bla*_KPC-2_, 6 *bla*_OXA-23-like_, 2 *bla*_OXA-23_, 4 *bla*_OXA-72_, 3 *bla*_OXA-58_, 1 *bla*_OXA-23-like_ plus *bla*_OXA-58_ and 1 *bla*_OXA-23-like_ plus *bla*_OXA-58_ plus *bla*_NDM_) and 17 non-CPE isolates (9 *bla*_ESBL_, 2 *bla*_pAmpC_, 4 *bla*_ESBL_ plus *bla*_pAmpC_ and 2 non-ESBL and non-pAmpC) gave negative results (Table 1 and Figure 3). Therefore, this method provided 100% sensitivity (95% CI of 92.6–100%) and 100% specificity (93.5–100%) for detecting the *bla*_OXA-48-like_ genes compared with the PCR-AGE method. The results could be easily interpreted within 3 to 5 min.

## 3. Discussion 

Currently, novel β-lactamase inhibitors have been available and used in combination with the old β-lactams as an alternative treatment for CRE or CPE infections. Ceftazidime combined with avibactam is recommended as the antimicrobial therapy option for infections caused by class A KPC- or class D OXA-48-like-producing Enterobacterales, whereas meropenem–vaborbactam or imipenem/cilastatin–relebactam is actively against CPE with class A carbapenemases, but not MBL or OXA-48-like producers [3,5,7,8]. OXA-48-like carbapenemases are now widely distributed among Enterobacterales with high prevalence in many countries [9]. We, therefore, developed the RPA-LF method for identification of the *bla*_OXA-48-like_ genes in Enterobacterales clinical isolates. 

In this study, the RPA method [22] was selected because it is carried out at lower and constant temperatures in less time than those of other isothermal amplification methods including loop-mediated isothermal amplification (LAMP) [20], helicase-dependent isothermal DNA amplification (HDA) [21], signal-mediated amplification of RNA technology (SMART) [23], cross priming amplification (CPA) [32] and multiple cross displacement amplification (MCDA) [33]. Primer design for the RPA method is also not complicated. From our experience, primers and probe for RPA-LF assay should be designed simultaneously. In addition, DNA samples can be extracted by a simple boiling method. It does not require the DNA purification and thermal cycler. The RPA reaction is carried out within 10 min at 37 °C. The RPA products can be visually detected by AGE, real-time fluorescent probes or an electrochemical biosensor [24,25]. However, it is noted that before being applied to the AGE assay, the RPA product should be purified [28] to eliminate the presence of lump of smear on the agarose gel, which is caused by proteins and crowding agents in the reagent kit. Incubation at 65 °C for 10 min after the RPA amplification step is recommended as an alternative purification step for the RPA-AGE assay [34,35]. In this study, the RPA products were visually inspected by the LF strip (within 5 min) because it is a simple and rapid device to clearly detect the RPA products. The RPA-LF assay can be performed within 15 min only, thus, being suitable at POC. The RPA-LF assay is now widely applied to detect and identify antimicrobial resistance genes, such as mobilized colistin resistance-1 (*mcr-1*) [26], macrolide efflux A (*mefA*) [27], methicillin resistance (*mecA*) [28], and extended-spectrum β-lactamase (ESBL) genes [29]. It was also successful to apply for rapid and direct detection of *Staphylococcus aureus* plus methicillin resistance as well as *Enterococcus* plus vancomycin resistance in positive blood cultures, suggesting the use of RPA-LF assay as a POC device [30,31]. The international patent for RPA primers of *bla*_OXA-48-like_ by Sutton et al. [36] was reported in 2017. Kuşkucu et al. [37] developed the RPA-LF assay for detecting *bla*_OXA-48-like_, whereas Wang et al. [38] also described the RPA-LF assay for identifying *bla*_KPC_, *bla*_NDM_, *bla*_OXA-48-like_ and *bla*_IMP_ in Enterobacterales with high sensitivity and specificity. However, small sample sizes of *bla*_OXA-48-like_-carrying isolates were included in both studies. 

We previously developed an isothermal amplification technique, in-house LAMP with hydroxynaphthol blue dye (HNB), for detecting *bla*_NDM_, *bla*_OXA-48-like_, *bla*_IMP-14_, *bla*_VIM_ and *bla*_KPC_ genes in Gram-negative bacilli [39]. This method is rapid, easy to perform, inexpensive, reliable and suitable for applying in a resource-limited setting. However, false-positive results with high rates were obtained from the detection of the *bla*_OXA-48-like_ genes by the LAMP-HNB assay due to the contamination of the LAMP products [40]. In the present study, we developed and evaluated the performance of the RPA-LF assay for the detection of *bla*_OXA-48-like_ genes from pure colonies. Our designed primers and probe were highly specific to the *bla*_OXA-48__-__like_ target. This was supported by the alignment of the *bla*_OXA__-__48__-__like_ variants showing no mismatch in both primers and probe. Their specificity, analyzed by using a nucleotide BLAST, also revealed no hit with other carbapenemase genes. Therefore, when tested with both *bla*_OXA-48__-__like_-positive and -negative isolates, it provided excellent sensitivity (100%) without any cross-reaction with other carbapenemase types (100% specificity). High sensitivity of the RPA-LF assay might also lead to the presence of false-positive results due to cross-contamination from previously obtained RPA products. In the present study, we found discrepancies between the first and second testing in one *bla*_OXA-48-like_-carrying *K. pneumoniae* isolate (1 from 61 isolates, 1.6%) and two isolates of *E. coli* and *K. oxytoca* with other *bla* genes (2 from 70 isolates, 2.9%). The modal results of these three isolates gave the same results as those of the gold standard method. To avoid contamination, TwistDx recommends that pre- and post-amplification steps, such as the master mix preparation, the addition of the sample DNA to the mixture, the RPA reaction, and the detection of the RPA product, should be performed in separate working areas [28]. In this study, the turnaround time of the RPA-LF assay is about 35 min including preparing the template or genomic DNA, performing the RPA reaction and detecting the RPA products on strip. However, preparation times for each step were not included. The RPA-LF assay also provided the limit of detection lower than those of the RPA-AGE and the conventional PCR-AGE method, similar to previous reports (10–100 times) [27,28,29,30,31]. To reduce the cost of the RPA-LF assay, the RPA reaction using the RPA reagents of TwistAmp™ Kit was performed in the small final volume of 12.5 µL as described previously [28,41]. The RPA products were then detected by half-cut commercial LF strip (Milenia Genline HybriDetect-1) [28]. Therefore, the RPA-LF assay is rapid, user-friendly, cost-effective and highly sensitive for *bla*_OXA-48-like_ detection in Enterobacterales.

Some limitations should be noted for this study: (i) among the *bla*_OXA-48-like_ group, only *bla*_OXA-48_ and *bla*_OXA-181_-carrying Enterobacterales isolates were obtained for testing the sensitivity, however the alignment of the *bla*_OXA-48-like_ variants revealed no mismatch in both primers and probe except for one mismatch from T to A bases (Table 2) in forward primer for *bla*_OXA-181_, *bla*_OXA-232_ and *bla*_OXA-484_; (ii) small samples or no samples of some carbapenemase gene types (e.g., *bla*_KPC_, *bla*_GES-2_, *bla*_GES-5_) for testing the specificity were included due to the low prevalence of these carbapenemase types in our area; (iii) molecular methods including the RPA-LF assay cannot differentiate true OXA-48-like carbapenemases from OXA-48-like variants lacking carbapenemase activity, such as OXA-163, OXA-247 and OXA-405; and (iv) the performance of this RPA-LF assay has not yet been evaluated in clinical samples, particularly blood samples. However, the developed RPA-LF assay is ready-to-use for differentiation of the *bla*_OXA-48-like_ in routine laboratories.

## 4. Materials and Methods

### 4.1. Bacterial Isolates 

A total of 131 Gram-negative bacilli isolates, 114 isolates carrying various carbapenemase genes and 17 isolates without carbapenemase genes, were used in this study (Table 1). Of the 114 isolates, 89 isolates were Enterobacterales carrying class A (4 isolates), B (26 isolates), D (60 isolates) and B plus D (1 isolate) genes, whereas 16 *Acinetobacter baumannii* with class D (15 isolates) and class B plus D (1 isolate), 1 *Acinetobacter pittii* with class D and 8 *P. aeruginosa* with class B genes were included for testing specificity (Table 1). They were collected between 2001 and 2020 from seven hospitals in different regions of Thailand [11]. The 17 Enterobacterales isolates included 15 isolates carrying other *bla* genes (9 *bla*_ESBL_, 2 *bla*_pAmpC_ and 4 *bla*_ESBL_ plus *bla*_pAmpC_) and obtained from our hospital between 2001 and 2015, and 2 non-*bla*_ESBL_ plus non-*bla*_AmpC_ reference strains (*E. coli* ATCC 25922 and *K. pneumoniae* ATCC BAA-1706). All isolates were identified by either conventional biochemical tests or VITEK®2 automated system (bioMérieux, Marcy-l’Étoile, France) and were of different strains by phenotypic and/or genotypic methods described elsewhere [42]. A *bla*_OXA-48_-harboring *K. pneumoniae* clinical isolate and *K. pneumoniae* ATCC BAA-1706 reference strain were used as *bla*_OXA-48_-positive and negative controls, respectively. 

The protocol of this study was approved by the Ethics Committee of Khon Kaen University (project number HE611605).

### 4.2. Genomic DNA Extraction 

Genomic DNA was extracted from pure culture of each test organism grown on Mueller Hinton agar (MHA) (Oxoid, Basingstoke, UK) at 37 °C overnight, by a simple boiling method for 10 min. After extraction, the bacterial suspension was centrifuged and the supernatant was used as a DNA template for conventional PCR and RPA-LF assays.

### 4.3. Conventional PCR Method 

Amplification of the *bla*_OXA-48-like_ genes by the PCR method of Poirel et al. [19] was used as a gold standard for evaluation of the RPA-LF assay. 

### 4.4. RPA Primer and Probe Design

**Figure 4:**
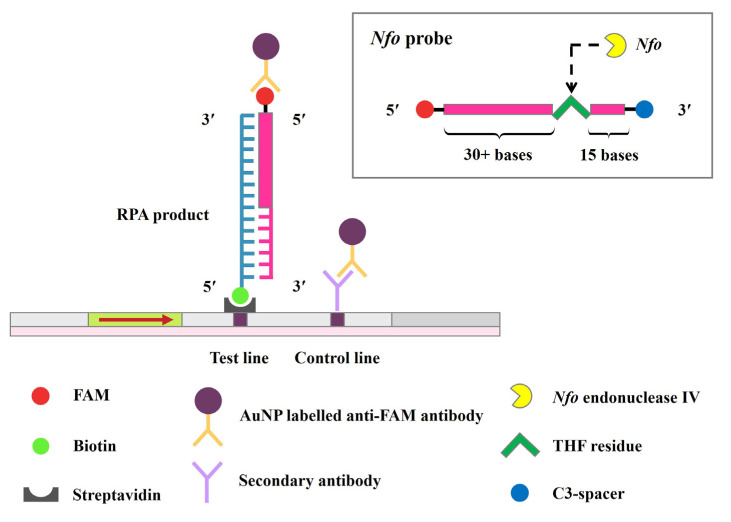
Schematic diagram of the RPA-LF assay: (i) the forward and reverse primers generate the biotin-labelled RPA products; (ii) the internal probe binds to the complementary DNA; (iii) *Nfo* endonuclease IV cleaves at a basic tetrahydrofuran (THF) site on the probe, thus, creating an extendable 3′-OH group for polymerization; (iv) the biotin- and FAM (carboxyfluorescein)-labelled RPA products are then detected by lateral flow strip based on sandwich-type reactions; (v) biotin binds to streptavidin on the test line, whereas FAM binds to the gold nanoparticle (AuNP) conjugated anti-FAM antibody; (vi) excess AuNP conjugated anti-FAM antibody is captured by the secondary antibody coated on the control line; and (vii) two visual bands of control and test lines indicate a positive result, whereas a visual band at control line only indicates a negative result.

Nucleotide sequences of the *bla*_OXA-48_ variants from GenBank® database (https://www.ncbi.nlm.nih.-gov/genbank/, accessed on 20 December 2019) were aligned using the Clustal Omega program (https://www.ebi.ac.uk/Tools/msa/clustalo/, accessed on 21 December 2019) to obtain the consensus sequence. Primers and probe specific to the *bla*_OXA-48_ group including *bla*_OXA-48_, *bla*_OXA-162_, *bla*_OXA-163_, *bla*_OXA-181_, *bla*_OXA-204_, *bla*_OXA-232_, *bla*_OXA-244_, *bla*_OXA-245_, *bla*_OXA-247_, *bla*_OXA-405_, *bla*_OXA-438_, *bla*_OXA-484_, *bla*_OXA-517_ and *bla*_OXA-519_ were manually designed based on the guidelines of TwistDx (Cambridge, UK) (Table 2). The 5′ ends of the reverse primer and the internal probe were antigenically labelled with biotin and carboxyfluorescein (FAM), respectively, to enable LF detection. A nucleotide of at least 30 bp far from the 5′ end and at least 15 nucleotides from the 3′ end was replaced by a basic tetrahydrofuran (THF) residue, where *Nfo* endonuclease IV cleaves when it binds to the complementary DNA, thus, creating an extendable 3′-OH group for polymerization. In addition, the 3′ end of the probe was labelled with C3-spacer, a polymerase extension blocking site, in order to prevent the extension of any un-hybridized probe (Figure 4). All designed RPA primers and probe were analyzed for their specificity by using a nucleotide BLAST search available through the National Center for Biotechnology Information (NCBI) website (https://blast.ncbi.nlm.nih.gov/Blast.cgi, accessed on 5 February 2020) and predicted for secondary structure and primer dimer by OligoEvaluator™ (http://www.oligoevaluator.com/LoginServlet, accessed on 5 February 2020) and for hetero-dimer by OligoAnalyzer tool (https://sg.idtdna.com/pages/tools/oligoanalyzer, accessed on 5 February 2020). The designed primers and probe were synthesized by Bio Basic Inc., Markham, Ontario, Canada. The primers were tested by both PCR-AGE and RPA-AGE reactions before further use. The PCR-AGE and RPA-AGE TwistAmp® Basic reaction components (Cambridge, UK) and conditions were performed as described previously [28].

### 4.5. RPA-LF and RPA-AGE Assays

The RPA-LF assay was optimized under various conditions compared with the RPA-AGE assay: amplification temperature at 25, 37, 39, and 42 °C for 20 min; and incubation time for 5, 10, 15, and 20 min at the optimal temperature obtained. Subsequent experiments were carried out under optimal conditions as described below.

The RPA-LF reaction with a final volume of 12.5 µL was performed using TwistAmp® nfo kit (TwistDx™, Cambridge, UK) with slight modification [28]. The RPA reaction mixture with a total volume of 45.5 µL was prepared as follows: 2.1 µL of 10 µM of OXA-48-F_RPA primer, 2.1 µL of 10 µM of OXA-48-R_RPA-LF primer, 0.6 µL of 10 µM OXA-48-probe, 29.5 µL of rehydration buffer, and 11.2 µL of sterile distilled water. The mixture was added with one lyophilized RPA reagent pellet, mixed by pipetting and divided equally (11.37 µL each) into four 0.2 mL microtubes. Subsequently, a 0.5 µL volume of DNA template was added to each tube. Then, each tube was added with 0.63 µL of 280 mM magnesium acetate (MgOAc), mixed well and incubated at 37 °C for 10 min. After the RPA amplification, the products were heated at 65 °C for 10 min to denature the proteins for protecting DNA-protein-crowding agent complexes from the RPA reaction [34,35]. The RPA products were analyzed by both LF strip (Milenia Genline HybriDetect-1, TwistDx, Cambridge, UK) and AGE methods. Briefly, 0.5 µL of RPA product was mixed with 49.5 µL of HybriDetect assay buffer (ratio of 1:100) in a 1.5 mL microcentrifuge tube. The LF strip was then immersed into the mixture and the bands were visualized within 3 to 5 min at room temperature. The presence of two red bands of the test and control lines indicated a positive result, whereas one red band of the control line only was considered negative. The appearance of the control line was indicative of the proper LF strip function. In addition, 1 µL of the RPA product was analyzed by AGE method.

### 4.6. Detection Limit of RPA-LF, RPA-AGE and PCR-AGE Assays

The detection limit of the RPA-LF assay was compared with that of the PCR-AGE method [19] used as a gold standard, and that of the RPA-AGE method. The bacterial suspension of the *bla*_OXA-48_-harboring *K. pneumoniae* control strain adjusted to yield turbidity of a 0.5 McFarland standard was serially 10-fold diluted from 10^0^ to 10^−8^. Bacterial colony count was performed by spread plate technique [43]. Briefly, a 100 µL volume of each bacterial dilution was spread on MHA and incubated overnight at 37 °C. Bacterial colonies were counted at the proper dilution containing 30 to 300 colonies and colony-forming unit (CFU)/mL was then calculated. Each dilution of the bacterial suspension was extracted and then used as a DNA template. The amplified *bla*_OXA-48_ products by the RPA-LF assay were compared with those by the RPA-AGE and PCR-AGE methods. The limit of detection was determined as the lowest DNA concentration that provided a positive result. 

### 4.7. Assessment of RPA-LF Assay

The performance of the RPA-LF assay was evaluated in 131 test isolates (Table 1) and compared to that of the PCR-AGE method. The experiments were carried out in duplicate. If any disagreement occurred, a third testing was performed and the modal result was used as the final result. The sensitivity, specificity and 95% confidence interval (CI) of the RPA-LF assay were calculated using the free software vassarStats (http://vassarstats.net/, accessed on 16 March 2021) [44].

## 5. Conclusions

Our RPA-LF assay is simple, rapid, reliable and cost-effective for detecting the *bla*_-OXA-48-like_ genes from pure colonies without the requirement of special equipment and expertise. It may be used as an alternative method for rapid identification of the *bla*_OXA-48-like_ genes at POC in areas with high prevalence of this carbapenemase type, leading to the selection of early and effective antimicrobial treatment for CPE infections.

## Figures and Tables

**Table 1:** Detection of the *bla*_OXA-48-like_ genes in Gram-negative bacilli by the RPA-LF assay compared with the conventional PCR method as a gold standard.

β-Lactamase Genes (n)	Species (n)	No. Isolates Tested by RPA-LF or PCR-AGE	
		Positive	Negative
Carbapenemase genes (114)			
Ambler class A (4)			
*bla*_KPC-2_ (1)	*K. pneumoniae* ATCC BAA-1705 (1)	-	1
*bla*_IMI-1_ (2) *bla*_IMI_ (1)	*E. cloacae* (2) *E. cloacae* (1)	- -	2 1
Ambler class B (32)			
*bla*_NDM_ (23)	*E. coli* (11)	-	11
	*K. pneumoniae* (8)	-	8
	*K. oxytoca* (1)	-	1
	*E. cloacae* (1)	-	1
	*C. freundii* (2)	-	2
*bla*_IMP-14a_ (3)	*P. aeruginosa* (2)	-	2
	*K. pneumoniae* (1)	-	1
*bla*_IMP_ (2)	*P. aeruginosa* (2)	-	2
*bla*_VIM-2_ (3)	*P. aeruginosa* (3)	-	3
*bla*_VIM_ (1)	*P. aeruginosa* (1)	-	1
Ambler class D (76)			
*bla*_OXA-48-like_ (51)	*K. pneumoniae* (45)	45	-
	*E. coli* (5)	5	-
	*E. cloacae* (1)	1	-
*bla*_OXA-48_ (4)	*K. pneumoniae* (3)	3	-
	*E. coli* (1)	1	-
*bla*_OXA-181_ (5)	*E. coli* (3)	3	-
	*K. pneumoniae* (1)	1	-
	*E. cloacae* (1)	1	-
*bla*_OXA-23-like_ (6)	*A. baumannii* (6)	-	6
*bla*_OXA-23_ (2)	*A. baumannii* (2)	-	2
*bla*_OXA-72_ (4)	*A. baumannii* (3)	-	3
	*A*. *pittii* (1)	-	1
*bla*_OXA-58_ (3)	*A. baumannii* (3)	-	3
*bla*_OXA-23-like_, *bla*_OXA-58_ (1)	*A. baumannii* (1)	-	1
Ambler class B and D (2)			
*bla*_NDM_, *bla*_OXA-48_ (1)	*K. pneumoniae* (1)	1	-
*bla*_NDM_, *bla*_OXA-23-like_, *bla*_OXA-58_ (1)	*A. baumannii* (1)	-	1
Non-carbapenemase genes (17)			
ESBL (9) *bla*_CTX-M-15_, *bla*_TEM_	*E. coli* (1)	-	1
*bla*_CTX-M-1-like_	*K. pneumoniae* (1)	-	1
*bla*_CTX-M-1-like_	*E. cloacae* complex (1)	-	1
*bla*_VEB-like_	*E. coli* (1)	-	1
*bla*_SHV_	*E. cloacae* complex (1)	-	1
*bla*_CTX-M-9_, *bla*_SHV-12_, *bla*_VEB-like_	*K. pneumoniae* (1)	-	1
*bla*_CTX-M-1-like_, *bla*_SHV_	*K. pneumoniae* (1)	-	1
*bla*_CTX-M-1-like_, *bla*_VEB_	*E. coli* (1)	-	1
*bla*_VEB_, *bla*_SHV_	*K. pneumoniae* (1)	-	1
pAmpC (2)			
*bla*_CMY-2_	*E. coli* (1)	-	1
*bla*_DHA-1_	*E. coli* (1)	-	1
ESBL and pAmpC (4)			
*bla*_CTX-M-1-like_, *bla*_CMY-2_	*E. coli* (1)	-	1
*bla*_CTX-M-15_, *bla*_CMY-2_	*E. coli* (1)	-	1
*bla*_VEB-like_, *bla*_CMY-8b_	*E. coli* (1)	-	1
*bla*_VEB-like_, *bla*_MOX-2-like_	*E. coli* (1)	-	1
Non-ESBL and non-pAmpC (2)	*E. coli* ATCC 25922 (1)	-	1
	*K. pneumoniae* ATCC BAA-1706 (1)	-	1
Total (131)		61	70

**Table 2:** Primers and probe used in this study for detection of the *bla*_OXA__-__48__-__like_ genes.

Assays	Primers	Sequences (5′→3′)	Product Sizes (bp)	Reference
PCR-AGE	OXA-48-F	GCGTGGTTAAGGATGAACAC	438	[19]
	OXA-48-R	CATCAAGTTCAACCCAACCG		
RPA-AGE	OXA-48-F_RPA	GATTATCGGAATGCCWGCGGTAGCAAAGGAATG	193	This study
	OXA-48-R_RPA	CAAGCTATTGGGAATTTTAAAGGTAGATGCGGG		This study
RPA-LF	OXA-48-R_RPA-LF	Biotin-CAAGCTATTGGGAATTTTAAAGGTAG ATGCGGG		This study
	OXA-48-probe	FAM-GTTGGAATGCTCACTTTACTGAACATA AAT-[THF]-ACAGGGCGTAGTTGT-C3-Spacer		This study

C3 Spacer, a polymerase extension-blocking site; FAM, carboxyfluorescein; THF, tetrahydrofuran; W, A or T nucleotide bases.

## Data Availability

Not applicable.
